# [Retracted] MicroRNA‑148a suppresses epithelial‑mesenchymal transition and invasion of pancreatic cancer cells by targeting Wnt10b and inhibiting the Wnt/β‑catenin signaling pathway

**DOI:** 10.3892/or.2023.8493

**Published:** 2023-02-06

**Authors:** Long Peng, Zhanying Liu, Jian Xiao, Yi Tu, Zhen Wan, Haiwei Xiong, Yong Li, Weidong Xiao

Oncol Rep 38: 301–308, 2017; DOI: 10.3892/or.2017.5705

Following the publication of this article, a concerned reader drew to the Editor's attention that various of the data panels showing the results from Transwell cell migration and invasion assay experiments in Figs. 2D and 4D contained groupings of cells that were markedly similar, even though the cells appeared in separate panels that were intended to show the results from different experiments. In addition, the cell images shown in Fig. 2B were strikingly similar to data that had appeared in different form in another article published by different authors at a different research institution.

After having conducted an internal investigation of this matter, the Editor of *Oncology Reports* has judged that the groupings of cells, appearing as they did among various different panels in Figs. 2 and 4, were too extensive that their apperance could have been attributed to pure coincidence. Also in view of the fact that some of the data were derived from a previously published source, the Editor has decided that this article should be retracted from the publication. After having been in contact with the authors of this study, they agreed with the Editor's decision to retract this article. The Editor sincerely apologizes to the readership for any incovenience caused, and we thank the reader for bringing this matter to our attention.

